# Validating RRP12 Expression and Its Prognostic Significance in HCC Based on Data Mining and Bioinformatics Methods

**DOI:** 10.3389/fonc.2022.812009

**Published:** 2022-02-01

**Authors:** Chao Wei, Ben Wang, Zhong-Huo Chen, Han Xiao, Lei Tang, Jia-Fu Guan, Rong-Fa Yuan, Xin Yu, Zhi-Gang Hu, Hua-Jun Wu, Zhi Dai, Kai Wang

**Affiliations:** ^1^ Hepato-Biliary-Pancreatic Surgery Division, Department of General Surgery, The Second Affiliated Hospital of Nanchang University, Nanchang, China; ^2^ Second Affiliated Hospital of Nanchang University, Jiangxi Province Key Laboratory of Molecular Medicine, Nanchang, China; ^3^ Second Affiliated Hospital of Nanchang University, Jiangxi Province Engineering Research Center of Hepatobiliary Disease, Nanchang, China; ^4^ Department of Hepato-Biliary-Pancreatic Surgery, Jiujiang First People’s Hospital, Jiujiang, China; ^5^ Second Affiliated Hospital of Nanchang University, Jiangxi Provincial Clinical Research Center for General Surgery Disease, Nanchang, China; ^6^ Liver Cancer Institute, Zhongshan Hospital, Fudan University, Shanghai, China

**Keywords:** RRP12, prognosis, hepatocellular carcinoma, tumor immune infiltration, molecule biomarkers

## Abstract

RRP12 (ribosomal RNA processing 12 homolog) is a nucleolar protein involved in the maturation and transport of eukaryotic ribosomal subunits and is a type of RNA binding protein. In recent years, considerable research has indicated that RRP12 is associated with the occurrence and development of multiple cancers. However, there is no research on RRP12 in hepatocellular carcinoma. Herein, we aimed to explore the role and significance of RRP12 in hepatocellular carcinoma.We used the TIMER and GEPIA databases to perform pan-cancer analyses of RRP12. The impact of RRP12 on the prognosis was analyzed through the GEPIA database. The relationship between RRP12 and immune cell infiltration was investigated by TIMER and GEPIA databases. Moreover, the expression of RRP12 in various liver cancer cells was evaluated by Western Blot to determine the cell line for the next experiment. Scratch test, Transwell test, and Edu tests were applied to validate the effects of RRP12 on the function of liver cancer cells. And the data were statistically analyzed.Pan-cancer analysis found that RPP12 was significantly upregulated in many cancers. Moreover, the prognostic analysis revealed that the difference in the expression of RRP12 has statistical significance for the overall survival rate and disease-free survival rate of liver cancer patients. In order to analyze the correlation between the expression level of RRP12 and clinical parameters, it was found that there was a significant negative correlation with tumor stage, tumor grade and tumor size. Univariate and multivariate analysis showed that RRP12 could be used as an independent prognostic factor for patients with hepatocellular carcinoma. Cellular experiments have proved that knocking down RRP12 can inhibit the proliferation, invasion, and metastasis of liver cancer cells.Therefore, RRP12 significantly affects the occurrence and development of HCC. Hence, RRP12 can become a potential target and prognostic biomarker for the treatment of hepatocellular carcinoma.

## Introduction

According to the latest cancer research report, hepatocellular carcinoma (LIHC) is the most common type of liver cancer. At the same time, hepatocellular carcinoma (LIHC) is the third most deadly cancer globally (1, 2). According to a new research report, there are more than 800,000 newly confirmed cases and 700,000 deaths from LIHC and related diseases yearly (3).Numerous inducements related to the development of HCC have been reported, including HBV virus infection (4), liver cirrhosis (5), excessive alcohol consumption (6), and immune imbalance (7). At present, sorafenib is a systematic therapy for patients with HCC (8). Although there has been some progress in diagnosis and therapy, the prognosis of HCC patients is still not satisfactory (9). Therefore, there is a great need to find suitable therapeutic targets and advanced biomarkers to improve the prognosis of HCC patients.

RRP12, also known as KIAA0609, is a protein involved in the maturation and export of ribosomal subunits. Although RRPl2 is a nuclear protein, it can participate in pre-transport and assembly of rRNA. Because the synthesis of ribosomes requires very high metabolic consumption, some scholars hypothesize that related proteins synthesized by ribosomes may participate in other biological processes in the cell to reduce costs. Moreover, studies have found that in yeast cells, RRP12 not only participates in the transport of 60S and 90S rRNA precursors but also physically interacts with Kapl21 and HRnr4 nucleoporins, thereby affecting the presence of ribonucleotide reductase subunits. The location of the cell nucleus further affects the transport of dNTPs in the nucleus and finally regulates the cell cycle or DNA damage response of yeast. However, in yeast cells, RRP12 is neither involved in the initiation process of the point protein complex nor the assembly and stabilization of the pre-replication complex. Furthermore, the total amount of ribosomes in yeast cells and the absence of RRP12 have no significant effect (10). In addition, studies have displayed that in osteosarcoma cells, RRP12 can enhance the resistance of tumor cells to chemotherapeutic drugs; after the expression of RRP12 was disturbed, the expression of p53 was significantly upregulated. Nonetheless, the mechanism of RRP12 in tumor cell resistance to chemotherapy drugs and regulating the expression of P53 has not been further studied (11). Recent researches have found that RRP12 is more highly expressed in colorectal cancer cells than in normal cells, and RRP12 can be used as a biomarker for the prognosis of colorectal cancer (12). Some studies have found that RRPl2 already has many functional characteristics in eukaryotes (13). A multitude of studies has proved that many proteins synthesized by ribosomes can participate in biological processes in cells. For example, 60S subunit synthetic factor Sdal can play a role in yeast cell cycle transition; Yph1/Nop7 and Noc3, two 60S subunit precursor particles, kick in the replication initiation complex and the entry in S phase (14, 15); RRP14 is used as a 60S precursor to synthesize protein and plays a close role in the process of mitotic spindle division (16). The 90S precursor synthetic subunits Utp6 and Utp7 play essential roles in centrosome replication and chromosome segregation, respectively (17, 18); Nop15, as a 60S subunit precursor factor, plays a vital role in the cytoplasmic division (19). The previous evidence indicates that RRP12 may have other biological functions besides ribosomal synthesis and may play a role in cellular proliferation, cycle, and DNA damage response. There are few studies on the role of RRP12 in tumorigenesis and development. In this study, through the expression and survival analysis of various human cancers, the differences in the expression and survival of RRP12 in HCC were identified. Then, the effects of RRP12 on the proliferation, invasion, and metastasis of hepatocellular carcinoma cells were explored. Through bioinformatics analysis, whether RRP12 is related to immune cell infiltration in liver cancer was investigated.

## Materials and Methods

### TIMER2.0 Database

TIMER2.0 (http://timer.cistrome.org/) database provides a friendly software interface and humanized visual interaction for researchers. To maximize the convenience for researchers to carry out the correlation between immune infiltration, gene expression, mutation, and survival characteristics in the TCGA database (20). Here, we used TIMER2.0 to explore the expression level of RRP12 between human cancer and matched normal tissues. Additionally, the database was used to analyze whether RRP12 was involved in immune cell infiltration and immune checkpoint in liver cancer.

### GEPIA Database

The GEPIA (http://gepia.cancer-pku.cn/index.html) database is a newly developed web server for cancer and normal gene expression profiling and interactive analysis. It fills the gap in cancer genomics extensive data information, thereby enabling clinical research enthusiasts to use public data resources more efficiently (21). Here, we used the GEPIA database to compare the expression level of RRP12 in human cancers that do not match the corresponding normal tissues in the TIMER2.0 database. Next, we analyzed the survival of statistically significant cancers in the TIMER2.0 and GEPIA databases, including two prognostic indicators of overall survival analysis (OS) and disease-free survival (RFS).). Finally, we used the GEPIA database to analyze whether RRP12 was associated with immune checkpoints in HCC.

### Cancer Gene Atlas (TCGA) Database

TCGA (https://cancergenome.nih.gov/) is a publicly available database that can improve diagnosis, treatment, and cancer prevention (22). The HCC transcriptome RNA sequencing data were downloaded from the database, including 374 HCC patients and 50 normal transcriptome data and corresponding clinical information. In order to avoid the influence of other factors, 26 HCC patients with incomplete clinical information in this database were ruled out. Data from 348 HCC patients were used for clinical correlation analysis and independent prognostic analysis. **Table 1** illustrates the basic characteristics of these 348 patients.

**Table 1:** Basic characteristics of 348 HCC patients.

Variables	HCC patient number
**Overall Survival Status(Alive/Dead)**	236/112
**Age(≤65/>65)**	226/122
**Gender(Female/Male)**	110/238
**Neoplasm Histologic Grade(I/II/III/IV)**	45/171/19/13
**T Stage(1/2/3/4)**	175/86/77/10
**N Stage(N0/N1-NX)**	256/92
**M Stage(M0/MI-MX)**	267/81
**Neoplasm Disease Stage(I/II/III/IV)**	173/84/86/5

### Functional Enrichment Analysis

The 50 genes (PCC>0.56) with the highest co-expression coefficients with RRP12 were identified through the GEPIA database. Then the R “ggplot2” package was utilized to perform enrichment analysis on these co-expressed genes, including GO and KEGG enrichment analysis.

### Cell Culture

HCC human cell lines HCCLM3, Huh-7, Hep3B, HepG2, MHCC97H and normal liver cell line 7702 were acquired from the Cell Bank of the Chinese Academy of Sciences (Shanghai, China). The frozen cells were thawed in a 37°C water bath and centrifuged for 3 minutes. The supernatant was discarded while the cell pellet was suspended. The resulting suspension was transferred to a cell flask and incubated at a constant temperature. Cellular adherence and growth were observed after 12 h. Afterward, the medium was discarded and the cells were rinsed with PBS. The cells were digested with trypsin solution to obtain the cell suspension. Next, the cell suspension was centrifuged and incubated at a constant temperature. After passaging, an appropriate amount of cells were taken, centrifuged, and the supernatant was discarded. Next, the cell pellet was suspended in a cryopreservation solution and transferred to a cryopreservation tube. Finally, the cryotube was placed in a gradient cryobox and left overnight in a refrigerator at -80 degrees celsius.

### Cell Transfection

Firstly, each well of a six-well plate was filled with 50000 cells, and INTERFER transfection reagent, Si-NC, and si-RRP12 were equilibrated at room temperature for 5 minutes. Lipofectamine™ 3000 Transfection Reagent (Invitrogen, Carlsbad, USA) was used following the manufacturer’s instructions. The protein was extracted after 48-72 h. WB was used to detect cell transfection efficiency, and follow-up experiments were performed (23).

### Western Blot Analysis

Total protein was prepared and western blot was performed according to the standard methods. The sample was electrophoresed, and transferred to the membrane with a constant current of 250mA. The membranes were blocked with 5% skimmed milk at room temperature for 1-3 hours and then incubated at 4°C overnight with the primary antibodies. After washing the membrane with TBST 3 times for 10 minutes each time, they were incubated with the secondary antibody at room temperature for 1h. Finally, pictures were taken in a dark room (24).

### Wound Healing Assay

First, marks were made on the back of the 6-wells plate and using a ruler, horizontal lines were evenly drawn at about every 0.5-1cm. The cells were added to a sterile environment and covered overnight. The following day, scratches were made perpendicular to the horizontal line on the back using the pipette’s tip. Subsequently, the cells were washed 3 times with PBS, the marked cells were removed, and a serum-free culture medium was added and placed in a 37°C incubator. Pictures were taken at 0 h, 24 h, and 48 h.

### Transwell Assay

The matrigel was melted at 4°C and diluted according to the volume ratio of matrigel: serum-free medium = 1:7, it was evenly mixed and spread on the upper layer of the transwell chamber; the matrigel-paved chamber was placed in the cell incubator for 3 hours and used after the matrigel solidified, and the transwell chamber was placed into a 24-well plate; 600ul cell culture medium containing 20% (v/v) FBS was added to the 24-well plate. 100ul serum-free cell suspension in the upper layer of the cell, containing 100,000 cells, was cultured in a cell incubator. The cell was cleaned, fixed, stained, and finally, pictures were collected under a microscope for analysis (25).

### EdU Assay

The cells used were from (Ribobio, Guangzhou, China). According to the instructions of the EDU staining kit, the EDU was diluted to 10 μmol/L using complete medium, 100 μl was then added to each well and incubated for 4 h. The medium was then discarded, washed with PBS twice and fixed with paraformaldehyde. Apollo staining was then added and cell proliferation was detected under a fluorescent microscope (26).

### Statistical Methods

R v3.6.0 (https://www.r-project.org/) and SPSS 26.0 (SPSS Inc., Chicago, IL) was used for all statistical analyses. Univariate Cox and multivariate Cox analyses were used for the independent prognostic analysis of RRP12. In this study, P<0.05 was considered to be statistically significant. Each experiment was repeated at least three times.

## Results

### Pan-Cancer Analysis of RRP12 Expression

**Figure 1:**
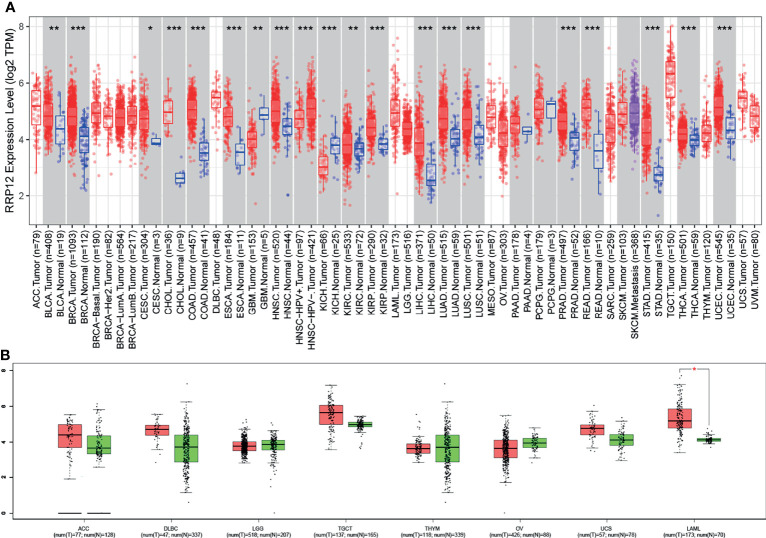
The transcriptional level of RRP12 in different types of cancer. **(A)** The transcriptional level of RRP12 in different types of cancer in the TIMER database. **(B)** Supplement to TIMER database based on GEPIA database. *p value < 0.05; **p value < 0.01; ***p value < 0.001.

The TIMER database showed that compared with normal samples, RRP12 was significantly upregulated in 17 cancers (p<0.05), including BLCA (urothelial carcinoma of the bladder), BRCA(invasive carcinoma of the breast),CESC (cervical cancer), CHOL (cholangiocarcinoma), COAD (colon adenocarcinoma), ESCA (esophageal cancer), HNSC (head and neck squamous cell carcinoma), KIRP (renal papillary carcinoma), KIRC (renal clear cell carcinoma), LIHC (hepatocellular carcinoma), LUAD (lung adenocarcinoma), LUSC (pulmonary squamous cell carcinoma), PRAD (prostate adenocarcinoma), READ (rectal adenocarcinoma), STAD (gastric cancer), THCA (thyroid cancer) and UCEC (endometrial cancer) (**Figure 1A**). However, in GBM (pleomorphic glioblastoma) and KICH (chromophobe cell carcinoma of the kidney), RRP12 was significantly down-regulated in both tumors compared with their corresponding normal samples (**Figure 1A**). Since some cancers did not possess corresponding normal samples in the TIMER database, we used the GEPIA database to supplement. The results showed that RRP12 was only significantly upregulated in LAML (acute myeloid leukemia) while not statistically significant in the others (p>0.05) (**Figure 1B**). This indicates that RRP12 may play an essential role in the occurrence and development of these 20 cancers.

### Prognostic Analysis of RRP12 in These 20 Cancers

**Figure 2:**
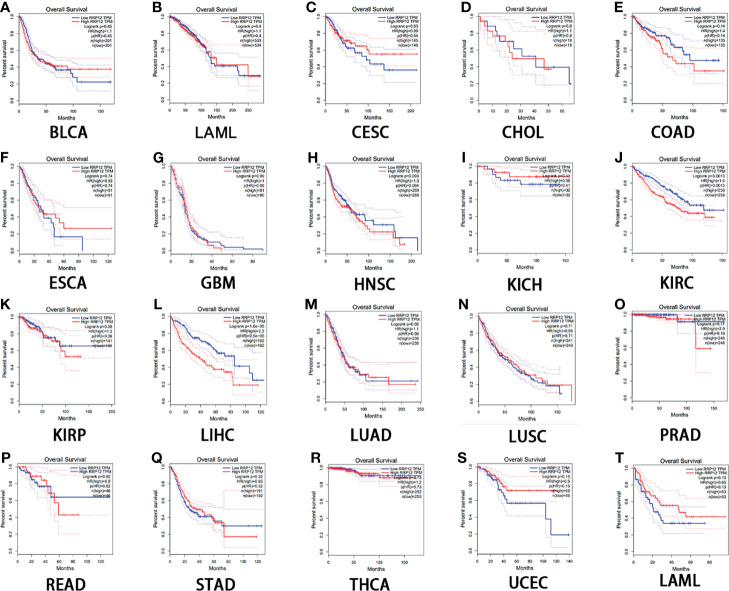
The overall survival (OS) analysis for RRP12 in various human cancers determined by GEPIA database. **(A–T)** The OS plot of RRP12 in BLCA **(A)**, BRCA **(B)**, CESC **(C)**, CHOL **(D)**, COAD **(E)**, ESCA **(F)**, GBM **(G)**, HNSC **(H)**, KICH **(I)**, KIRP **(J)**, KIRC **(K)**, LIHC **(L)**, LUAD **(M)**, LUSC **(N)**, PRAD **(O)**, READ **(P)**, STAD **(Q)**, THCA **(R)**, UCEC **(S)** and LAML **(T)**. p<0.05 is statistically significant.

**Figure 3:**
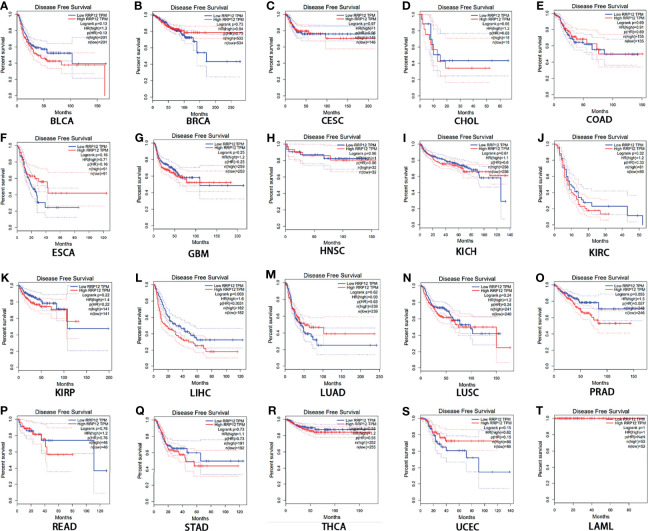
The disease-free survival (RFS) analysis for RRP12 in various human cancers determined by GEPIA database. **(A–T)** The RFS plot of RRP12 in BLCA **(A)**, BRCA **(B)**, CESC **(C)**, CHOL **(D)**, COAD **(E)**, ESCA **(F)**, GBM **(G)**, HNSC **(H)**, KICH **(I)**, KIRP **(J)**, KIRC **(K)**, LIHC **(L)**, LUAD **(M)**, LUSC **(N)**, PRAD **(O)**, READ **(P)**, STAD **(Q)**, THCA **(R)**, UCEC **(S)** and LAML **(T)**. p<0.05 is statistically significant.

Then, we used GEPIA to analyze the survival of RRP12 in these 20 cancers. For overall survival (OS), the results showed that the high expression of RRP12 is negative correlation with KIRC (clear cell carcinoma of the kidney) and LIHC (hepatocellular carcinoma) and the correlation had a poor prognosis (P<0.05), and the rest were not statistically significant (**Figure 2**). For disease-free survival (RFS), the results showed that only the high expression of RRP12 in LIHC (hepatocellular carcinoma) had a poor prognosis (p<0.05), and the rest were not statistically significant (**Figure 3**). Finally, by combining OS and RFS, RRP12 can be used as a marker for the poor prognosis of HCC patients.

### Clinical Relevance Based on the TGCA Database

**Figure 4:**
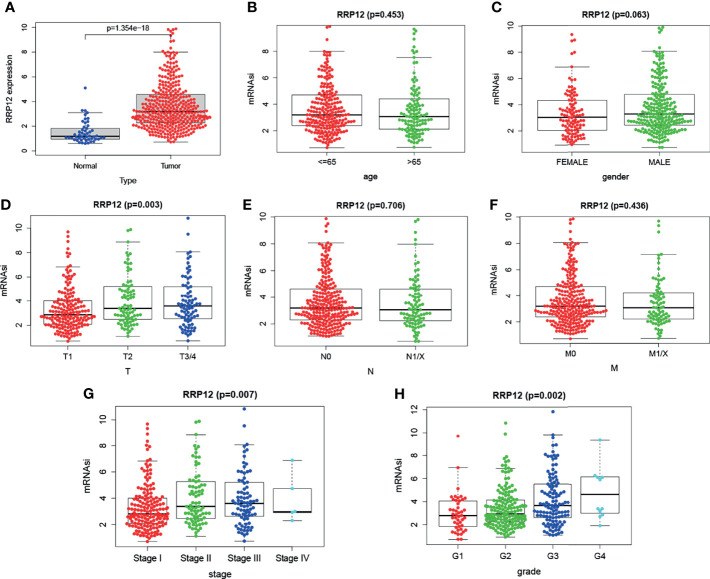
The analysis of RRP12 in TGCA. **(A)** The analysis of the expression between tumor tissue and normal tissue. The results show that RRP12 is highly expressed in tumors. **(B–H)** The relationship between the expression of RRP12 and the clinicopathological parameters of HCC patients. p<0.05 is statistically significant.

We downloaded transcriptome data and clinical data of 374 cases of liver cancer patients and 50 cases of normal or adjacent cancers from TGCA. First, we used the R “beeswarm” package to study whether the expression of RRP12 was different in tumor samples and normal or adjacent samples. The results showed that RRP12 was significantly high in tumor samples (**Figure 4A**), consistent with the results from the Unanimous TIMER database. Then, 7 clinical indicators, including age, gender, tumor grade, tumor stage, TNM stage, and RRP12 expression, were analyzed, and the results showed that the expression of RRP12 was significantly positively correlated with T stage, tumor stage, and tumor grade (p< 0.05), whereas the other clinical indicators were not statistically significant (**Figures 4B–H**).

### Functional Enrichment Analysis

**Figure 5:**
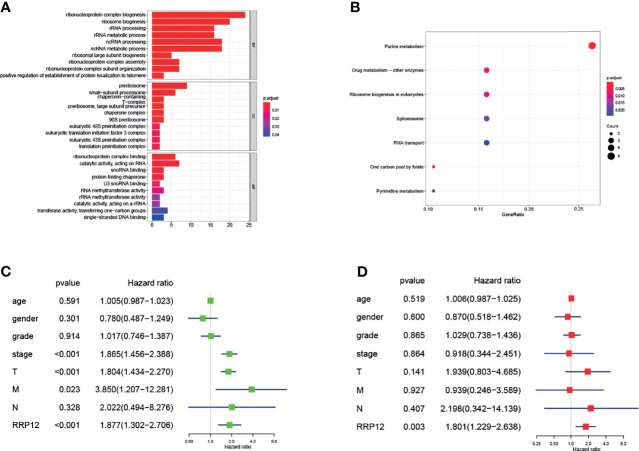
**(A, B)** The 50 genes with the highest co-expression coefficients with RRP12 are found through the GEPIA database (PCC>0.56), and then these 51 genes are enriched by GO and KEGG through the R “ggplot2” package. **(C, D)** It is a univariate and multivariate Cox regression analysis of the association between 7 clinicopathological factors and the overall survival rate of patients.

Find the 50 genes most closely related to RRP12 in liver cancer through the GEPIA database. Enrichment analysis of these genes co-expressed with RRP12 showed that in GO enrichment, the main functional pathways were “ribonucleoprotein complex biogenesis”, “ribosomal biogenesis”, “rRNA processing” and “rRNA metabolic process”, “Pre-ribosome” and “ribonucleoprotein complex binding” (**Figure 5A**). In KEGG enrichment, the main enrichment pathways were the “purine metabolism” and “pyrimidine metabolism” (**Figure 5B**). So we concluded that the co-expressed genes of RRP12 in liver cancer were mainly related to rRNA processing and metabolic pathways.

### Independent Prognostic Analysis of RRP12 in HCC

We have previously concluded that RRP12 was significantly negatively correlated with OS and RFS in HCC patients. According to univariate analysis, tumor stage, T stage, and RRP12 were related to a shorter survival time of patients, while the other clinical indicators were not statistically significant (**Figure 5C**). Later, we conducted multivariate analysis and derived that RRP12 were related to a shorter survival (**Figure 5D**). Based on the previous observations, we can conclude that RRP12 can be used as an independent prognostic factor for HCC patients.

### The Role of RRP12 in Immune Cell Infiltration in HCC

**Figure 6:**
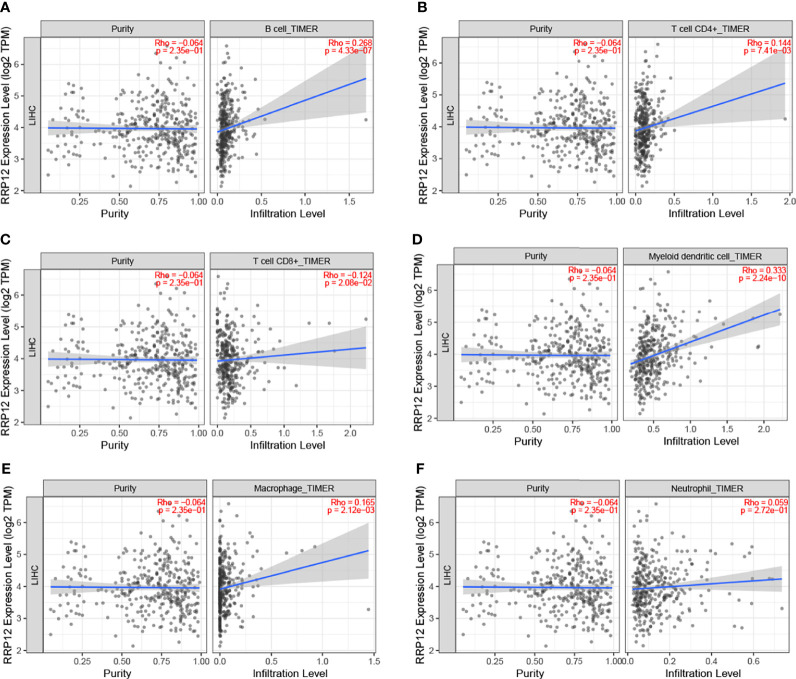
The relationship of immune cell infiltration with RRP12 expression level in HCC. The correlation of RRP12 expression level with B cell **(A)**, CD8+ T cell **(B)**, CD4+ T cell **(C)**, dendritic cell **(D)**, macrophage **(E)**, and neutrophi **(F)** infiltration level in HCC.

In order to study the possible functions and mechanisms of RRP12, we analyzed the relationship between RRP12 and six critical immune cells through the TIMER database, including B lymphocytes, CD8 + T lymphocytes, CD4 + T lymphocytes, macrophages, neutrophils, and dendritic cells. The results showed that the expression of RRP12 was significantly correlated with B lymphocytes, CD8 + T lymphocytes, CD4 + T lymphocytes, macrophages, and dendritic cells in HCC (P<0.05), among which B lymphocytes and dendritic cells were significantly correlated, and the correlation between RRP12 expression level and macrophages was not statistically significant (**Figure 6**). Therefore, we speculate that RRP12 may affect the occurrence and development of liver cancer through immune cells.

### The Role of RRP12 in Immune Escape of HCC

**Figure 7:**
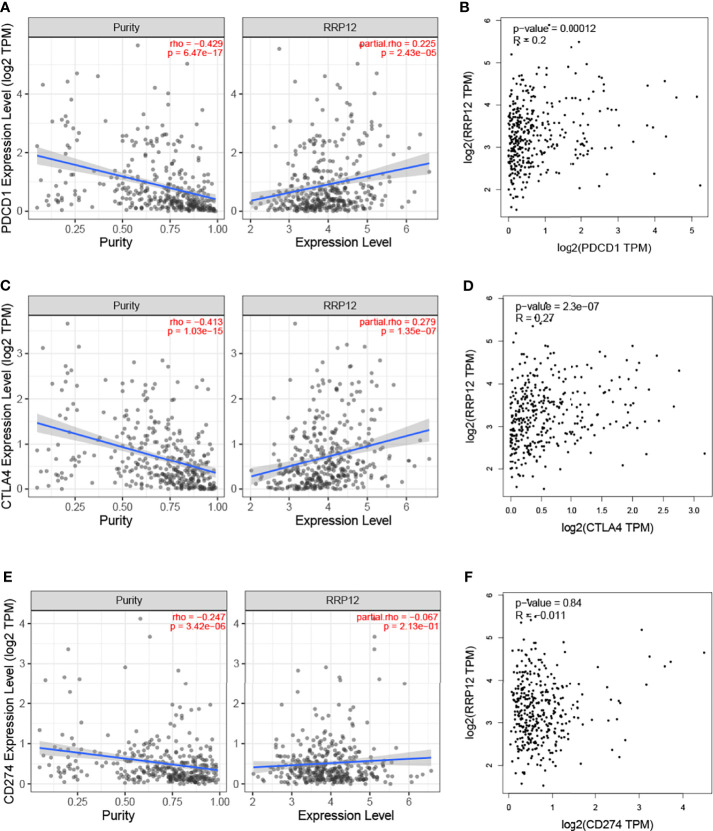
Correlation of RRP12 expression with PD-1, PD-L1, and CTLA-4 expression in HCC. **(A)** Spearman correlation of RRP12 with expression of PD-1 in HCC adjusted by purity using TIMER. **(C)** Spearman correlation of RRP12 with expression of CTLA-4 in HCC adjusted by purity using TIMER. **(E)** Spearman correlation of RRP12 with expression of PD-L1 in HCC adjusted by purity using TIMER. Spearman correlation of RRP12 with expression of CTLA-4 in HCC adjusted by purity using TIMER. **(B)** The expression correlation of RRP12 with PD1 in HCC determined by GEPIA database. **(D)** The expression correlation of RRP12 with CTLA-4 in HCC determined by GEPIA database. **(F)** The expression correlation of RRP12 with PD-L1 in HCC determined by GEPIA database.

There are three immune checkpoints related to immune escape in HCC, namely PD1, PD-L1, and CTLA-4. In order to analyze the value of RRP12 in the occurrence and development of HCC, we first studied the relationship between RRP12 and the molecular markers PDCD1, CD274, and CTLA4 corresponding to PD1, PD-L1, and CTLA-4 through the TIMER database. The results revealed that the expression level of RRP12 was significantly positively correlated with PDCD1 and CTLA4 in HCC, adjusted by purity (**Figures 7A, C, E**). Through the analysis of the GEPIA database, the results showed that RRP12 was also significantly positively correlated with PDCD1 and CTLA4 in HCC (**Figures 7B, D, F**). The above results suggest that RRP12 may affect the occurrence and development of HCC through tumor immune escape.

### Downregulating the Expression Level of RRP12 Inhibits the Proliferation, Invasion, and Migration of HCC

**Figure 8:**
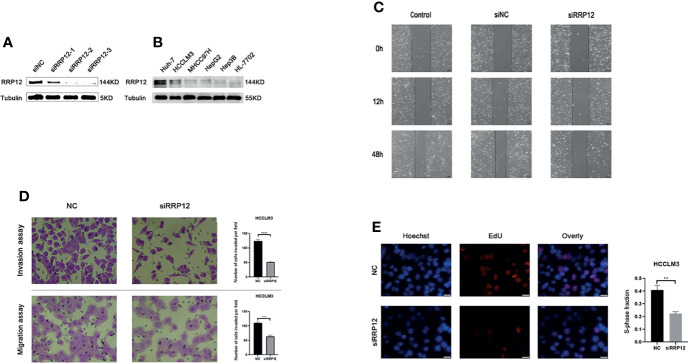
**(A)** Knockdown the expression of RRP12 in HCCLM3 cells, and NC was the negative control group. **(B)** The expression of RRP12 in Hepatocellular carcinoma Cell Lines and normal liver cell line.HCCLM3 and Huh-7 cells are higher than other HCC cells. **(C)** The scratch distance of the siRRP12 group did not change significantly, while the scratch distance of the control and siNC group was reduced significantly. It showed that Knockdown of RRP12 expression inhibits HCC invasion and migration *in vitro*. **(D)** The number of cells in the siRRP12 group was less than the NC group in the transwell assay (p<0.01), and the NC group was the negative control group. **(E)** Knockdown of RRP12 expression inhibits HCC proliferation *in vitro*. The number of proliferating cells in the siRRP12 group was less than that in the NC group, and the NC group was the negative control group.

Earlier, we found that the high expression of RRP12 was significantly positively correlated with tumor grade, stage, and T stage of HCC patients. From this, we can infer that the expression of RRP12 may be related to the proliferation, invasion, and metastasis of HCC. Then, we verified this inference through cell function experiments. The WB experiment was carried out to compare the expression level of RRP12 in a variety of HCC cells. The results showed that RRP12 was expressed higher in HCCLM3 and Huh-7 cells than in other HCC cells (**Figure 8B**). We used WB experiments to corroborate that interference fragments reduced the expression of RRP12 in HCCLM3 cells. The results confirmed that RRP12 was significantly decreased in HCCLM3, and NC was the negative control group (**Figure 8A**). In order to verify the effect of RRP12 on the metastatic ability of HCC, through the scratch experiment on HCCLM3, the scratch spacing of the NC group was significantly reduced after 48 hours, whereas the scratch spacing of the RRP12 knockdown group did not change significantly, so we concluded that the cancer cell invasion and migration ability decreased (**Figure 8C**). Similarly, the Transwell assay displayed that the number of cells in the RRP12 knockdown group was significantly smaller than that in the NC group, and the ability of cancerous cell invasion and migration decreased (**Figure 8D**). The Edu experiment showed that the cell proliferation ability of the RRP12 knockdown group was significantly lower than that of the NC group (**Figure 8E**).

## Discussion

There are basically no symptoms in early HCC. When patients are diagnosed, most of them may have reached a locally advanced stage or involve distant metastases, which dramatically affects the treatment (27). Related literature has reported more than 200 genes related to the prognosis of HCC. However, existing biomarkers and therapeutic targets are far from enough (28). Therefore, exploring biomarkers for early diagnosis of HCC can lead to better treatment and prognosis for patients.

Numerous molecules in the RRP family can be used as tumor markers. For example, RRP15 can inhibit the growth of liver cancer cells by inducing senescence and apoptosis (29); RRP1B can upregulate the expression of claudin-1 by depleting DOCK1, and increase cell viability and motility of low-density breast cancer cells (30); RRP8 can be used as a novel biomarker for triple-negative breast cancer (31). Herein, we evaluated the effects of RRP12 on HCC. First, we conducted a pan-cancer analysis of RRP12 through the TIMER database and supplemented the research with the GEPIA database. Then, the survival analysis (including overall survival and disease-free survival) of those cancers with different expression levels of RRP12 showed that the high expression of RRP12 in HCC patients was associated with a poor prognosis, and the results suggest that RRP12 can be used as a prognostic molecule for HCC. Later, 374 HCC patients and 50 normal transcriptome data and corresponding clinical information were downloaded from the Cancer Gene Atlas (TCGA) database. Through these data, the relationship between the expression of RRP12 and seven clinical parameters was analyzed, and it was observed that RRP12 was significantly positively correlated with tumor grade, stage, and T stage. Moreover, it was speculated that RRP12 might be related to tumor proliferation, invasion, and metastasis. Univariate and multivariate analysis showed that RRP12 could also be used as an independent prognostic factor for HCC. These studies indicate that RRP12 may be used as a novel biomarker for HCC.

The enrichment analysis of GO and KEGG showed that RRP12 might be mainly related to rRNA processing and metabolism. Moreover, multiple studies have highlighted that affecting the biosynthesis and metabolism of ribosomes can affect the occurrence and development of tumors. For example, pRb and p53 can inhibit the growth of breast cancer cells by inhibiting rRNA processing (32). Dyskerin is an essential nucleolar protein that can affect the growth of tumors through its related mechanism of rRNA processing in the precursor (33); the rRNA synthesis inhibitor CX-5461 can induce acute lymphocytes by activating the ATM/ATR pathway, leukemia cells stagnate and die (34). The growth of triple-negative breast cancer requires high-demand ribosome biosynthesis (35); ribosome biogenesis can affect the biological behavior of cell senescence and cancer through a connection with the target of the rapamycin (TOR) signaling pathway (36). The above evidence indicates that RRP12 can also affect the occurrence and development of tumors by affecting the synthesis and metabolism of ribosomes.

A large number of studies have reported that tumor immune cell infiltration can affect the effects of chemotherapy, radiotherapy, and immunotherapy in cancer patients, thereby influencing the prognosis of patients (37–41). Our research found that the expression of RRP12 has a significant correlation with B lymphocytes, CD8 + T lymphocytes, CD4 + T lymphocytes, macrophages, and dendritic cells in HCC. This can also indicate that RRP12 may affect the occurrence and development of HCC through immune infiltration. In addition, the infiltration of immune cells into the tumor microenvironment is not enough, and immune checkpoints need to be fully expressed in the tumor microenvironment so that immunotherapy is most effective (42). Therefore, our study also analyzed the relationship between RRP12 and immune checkpoints and found that RRP12 has a significant positive correlation with PD-1 and CTLA-4 in HCC. We contemplate that RRP12 can be used as a target for HCC immunotherapy.

In order to verify the conclusions of bioinformatics, we carried out WB and a series of functional experiments. The WB experiment revealed that RRP12 was significantly upregulated in HCCLM3. Scratch experiments showed that RRP12 could promote the transfer of HCC cells. Transwell experiments proved that RRP12 could promote the invasion and migration of HCC cells. Likewise, the Edu experiment proved that RRP12 could promote the proliferation of HCC cells.

In short, through our research, we can find that RRP12 can be used as a prognostic marker and a new therapeutic target for HCC. However, this study still has certain limitations. We have neither conducted *in vitro* experiments to verify the effect of RRP12 on HCC nor have we researched the mechanism of RRP12 on the occurrence and development of HCC. In the future, we will improve RRP12’s research on HCC.

## Data Availability Statement

The datasets presented in this study can be found in online repositories. The names of the repository/repositories and accession number(s) can be found in the article/supplementary material.

## Author Contributions

WC, BW, Z-HC, and KW conceived and designed the study. WC, BW, Z-HC, HX, LT, J-FG, and KW performed experiments. WC, BW, Z-HC, and KW contributed to Materials and methods. HX, LT, R-FY, XY, Z-GH, and ZD analyzed the data. WC, BW, Z-HC, and KW wrote the manuscript. All authors read and approved the final manuscript.

## Funding

The National Natural Science Foundation of China (82060454),the key research and development program of Jiangxi Province of China(20203BBGL73143) supported this study.

## Conflict of Interest

The authors declare that the research was conducted in the absence of any commercial or financial relationships that could be construed as a potential conflict of interest.

## Publisher’s Note

All claims expressed in this article are solely those of the authors and do not necessarily represent those of their affiliated organizations, or those of the publisher, the editors and the reviewers. Any product that may be evaluated in this article, or claim that may be made by its manufacturer, is not guaranteed or endorsed by the publisher.
